# Application of Normal-Phase Silica Column in Hydrophilic Interaction Liquid Chromatography Mode for Simultaneous Determination of Underivatized Amino Acids from Human Serum Samples via Liquid Chromatography–Tandem Mass Spectrometry

**DOI:** 10.3390/cimb45120586

**Published:** 2023-11-22

**Authors:** Krisztina Németh, Ildikó Szatmári, Viktória Tőkési, Pál Tamás Szabó

**Affiliations:** 1 MS Metabolomics Research Laboratory, Centre for Structural Science, Research Centre for Natural Sciences, Hungarian Research Network, Magyar Tudósok Krt. 2, H-1117 Budapest, Hungary; nkriszti94@gmail.com; 2 Institute of Chemistry, Eötvös Loránd University, Pázmány Péter u. 1/A, H-1117 Budapest, Hungary; 3 Department of Pediatrics, Semmelweis University, Bókay János u. 54, H-1083 Budapest, Hungary

**Keywords:** amino acids, LC-MS/MS, normal-phase chromatography, standard addition calibration

## Abstract

In neonatal screening, amino acids have a significant diagnostic role. Determination of their values may identify abnormal conditions. Early diagnosis and continuous monitoring of amino acid disorders results in a better disease outcome. An easy and simple LC-MS/MS method was developed for the quantitation of underivatized amino acids. Amino acids were separated using a normal-phase HPLC column having a totally porous silica stationary phase and using classical reversed-phase eluents. Mass spectrometry in multiple reaction monitoring mode was used for the analysis, providing high selectivity and sensitivity. A standard addition calibration model was applied for quantitation using only one isotope-labeled internal standard for all amino acids. Five calibration points were used for quantitation, and the method was successfully validated. The slopes of the calibration curves of the individual amino acids in parallel measurements were found to be similar. Since the measured slopes were reproducible, one serum sample could represent every series of serum samples of a given day. The method was tested on human serum samples and adequate results were obtained. This new method can be easily applied in clinical laboratories.

## 1. Introduction

Amino acids (AAs) are the building blocks of proteins and are very important for all living organisms. AAs play an important role as neurotransmitters and precursors for the biosynthesis of hormones, glycolipids and nucleic acids [1,2]. AA analysis is essential in clinical biochemistry for diagnosis and monitoring of metabolic diseases (like phenylketonuria, maple syrup urine disease and diabetes) [3,4]. The introduction of triple quadrupole tandem mass spectrometry (MS/MS) in clinical laboratories revolutionized the ability to carry out newborn screening (NBS) for multiple diseases in a single multiplexed analysis using dried blood spots (DBSs) [5]. The semiquantitative amino acid and acylcarnitine profile obtained via MS/MS is the most universal platform currently available for the analysis of metabolites in DBSs for applications in newborn screening. The screen positives need to undergo selected confirmatory analyses and clinical examination. In the diagnosis and clinical follow-up of inherited metabolic disorders, the quantitative analysis of AAs is indispensable; however, it is often overlooked due to difficulties in obtaining reliable data.

There are several analytical techniques for the detection and quantitation of AAs, like ultraviolet detection [6,7,8], fluorescence detection [8,9,10], charged aerosol detection [11,12], electrochemical detection [13,14] or mass spectrometry [15,16,17,18,19]. The analysis is often performed after chemical derivatization. The gold standard for AA analysis was invented a few decades ago, using post-column derivatization with ninhydrin after ion-exchange chromatography [20]. Most pre-column derivatization techniques rely on reversed-phase liquid chromatography (RP-LC) separation using several derivatization agents, such as ortho-phthalaldehyde [21]. Often, derivatization methods are not convenient and are usually time-consuming. Underivatized, free AAs have no chromophore groups, making classical ultraviolet detection impossible. Hence, AAs are mainly measured by means of liquid chromatography coupled with tandem mass spectrometry (LC-MS/MS), providing high selectivity and sensitivity. This detection technique is commonly used with RP-HPLC; however, it is not suitable for extremely polar AAs due to low retention. Ion-pair modifiers combined with reversed-phase chromatography provide better retention for the polar components; however, the MS system often gets contaminated due to ion-paring agents [22]. Another possible solution to improve the retention of polar components like free AAs is hydrophilic interaction liquid chromatography (HILIC). HILIC has become popular in the last decade, facilitating the retention and separation of very polar components. Like HILIC, classical normal-phase (NP) chromatography uses polar stationary phases such as amino, cyano or silica. Polar components have strong electrostatic binding to the stationary phases, resulting in longer retention times. Mobile phase pairs, such as methanol/water or acetonitrile/water, can be applied in the HILIC mode, which are commonly used in reversed-phase chromatography. However, HILIC often requires buffers, which cause ion suppression, and the methods need a longer equilibration time [23,24]. The need for quantitating polar target molecules pushed our interest toward HILIC-type separations. A series of classical HILIC columns and normal-phase stationary phases were tested and found that totally porous silica particles gave stable and reliable retentions and symmetric peak shapes for polar compounds. Rx-SIL normal-phase column was successfully applied in our previous work for the separation and quantitation of free nucleic acid bases [25]. This column was tested on underivatized amino acids, and excellent retentions were found for all of the free amino acids tested.

Isotope-labeled AAs are used for accurate quantitation as internal standards. These labeled molecules have very similar chromatographic and mass spectrometric behavior as the analytes, like retention time or ionization efficiency. Furthermore, isotope-labeled standards can eliminate problems due to analyte loss during sample preparation [26,27].

Clinical laboratories rarely perform method development, and, typically, simple and relatively inexpensive solutions are preferred. Few commercial kits are available for quantitative amino acid analysis [28,29]; however, they are expensive, and like other derivatization techniques, sample preparation is time-consuming, standards must be freshly prepared and chemical modifications can cause errors, like by-product formation [30].

The aim of this study was to develop a sensitive, robust, accurate and easily adoptable method for the quantitation of AAs without derivatization, which can be applied in metabolic investigations for inherited metabolic disorders. We attempted to avoid using ion-pairing agents or long equilibration times. Finally, an MS friendly method was developed using a normal-phase column in HILIC mode using classical RP eluents. A standard addition quantitation model was applied, handling the matrix effect in the most realistic way. Furthermore, we kept the calibration protocol for the daily measurements short. Only one isotope-labeled AA (glutamic acid-2,3,3,4,4-d5) was used for the quantitation, reducing the costs of the measurement while keeping the precision of the method. The method was validated and used for the quantitation of AAs in human serum samples.

## 2. Experimental

### 2.1. Materials and Reagents

An amino acid mix solution (consisting of 17 AAs), asparagine, glutamine and tryptophan were purchased from Supelco (Merck KGaA, Darmstadt, Germany). 4-aminobutyric acid-2,2-d2 and glutamic acid-2,3,3,4,4-d5 were purchased from Sigma-Aldrich (Merck KGaA, Darmstadt, Germany). Acetonitrile (MeCN, gradient grade) and formic acid (FA, >99%) were obtained from VWR International (Radnor, PA, USA). Water was produced by a MilliQ Purification System from Millipore (Merck KGaA, Darmstadt, Germany). Hank’s Balanced Salt Solution (HBSS) was obtained from Thermo Fisher Scientific (Waltham, MA, USA).

### 2.2. Instrumentation and Conditions

A vortex mixer (VELP Scientifica Srl, Usmate, Italy) and a microcentrifuge (Thermo Fisher Scientific, Waltham, MA, USA) were used for sample preparation. A QTRAP 6500 triple quadrupole linear ion trap mass spectrometer equipped with a Turbo V Source (Sciex, Framingham, MA, USA) and an Agilent 1100 Series HPLC (Agilent, Santa Clara, CA, USA) was used for LC-MS/MS analysis. Data acquisition was performed using Analyst software 1.6.3, and the data were analyzed using MultiQuant software 2.1 (Sciex, Framingham, MA, USA). Chromatographic separation was carried out using an Agilent Zorbax Rx-SIL column (250 mm × 4.6 mm, 5 µm, Agilent, CA, USA). Water containing formic acid in 0.1 *v*/*v*% (eluent A) and acetonitrile containing formic acid in 0.1 *v*/*v*% (eluent B) were used for the separation. The flow rate was 1 mL/min and 5 µL of the samples were injected. The column temperature was ambient, and the samples were kept at 10 °C in the autosampler during the acquisition. A segmented gradient method was used, and the initial eluent composition was 25% “A” eluent and 75% “B” eluent. This composition was kept for 1 min and a 4 min linear gradient was applied to reach 45% A. In the next segment, the composition of A was increased to 80% in 2 min and this composition was held for 2 min. The initial composition (25% A) was reached in 0.5 min, which was followed by a 5.5 min equilibration part.

Electrospray ionization was performed in the positive-ion mode. The optimized source conditions for the mass spectrometric measurements were as follows: spray voltage of 5000 V; evaporation temperature of 450 °C; and curtain, evaporation and drying gases were 35, 40 and 40 psi, respectively. The MS/MS was operated under multiple reaction monitoring (MRM) mode with nitrogen as the collision gas set at “medium”. The MRM transitions and analyzer parameters can be found in Table 1. The dwell time for each MRM transition was set at 20 msec.

### 2.3. Biological Samples

The biological samples were human serum samples from non-metabolic and inherited metabolic disorder patients (newborns and children) obtained from the Department of Pediatrics (Semmelweis University, Hungary).

Ethical Committee approval was not necessary for this study because this was a secondary data analysis. The samples were anonymized before arrival at the research laboratory, no record of patient information was known, and no interaction occurred between the individuals and the researchers.

### 2.4. Preparation of Stock Solutions and Calibration Standards

The concentration of AAs in the commercially available standard mix was 1.25 mM for cystine and 2.5 mM for all the others, including alanine, arginine, aspartic acid, glutamic acid, glycine, histidine, isoleucine, leucine, lysine, methionine, phenylalanine, proline, serine, threonine, tyrosine and valine. Asparagine, glutamine and tryptophan were prepared by dissolving the solid AAs in pure water with a concentration of 25 mM. These 3 AAs had to be added to the standard mix solution to have the final concentration of each AA at 100 µM, except for cystine, where the concentration was 50 µM. This stock solution was further diluted with water for the standard addition experiment, resulting in AA working solutions with concentrations of 1, 2, 4, 8 and 16 µM, respectively.

Glutamic acid-2,3,3,4,4-d5 (d5-Glu) was used as the internal standard (IS) in a concentration of 2 µM.

The calibration standards for standard addition were prepared from human serum samples in 3 steps, resulting in Solution *A*, *B* and *C* series, respectively.

Solution *A*: 50 µL of serum sample was diluted with 450 µL of water.

Solution *B*: 25 µL of Solution *A* was mixed with 375 µL of water, 50 µL of IS and 50 µL from each concentration level of the AA working solution (1, 2, 4, 8 and16 µM), resulting in five different Solutions *B*.

Solution *C*: 300 µL of acetonitrile was added to 100 µL of each Solution *B* (to precipitate proteins from serum), resulting in five different Solutions *C*. These mixtures were vortexed and centrifuged for 5 min at 13,300 rpm, and 100 µL from each supernatant was transferred into vials. Then, 5 µL of the samples was injected for LC-MS/MS measurements.

### 2.5. Preparation of Biological Samples

Human serum samples were used after the method validation. A total of 50 µL of serum was diluted with 450 µL of water, resulting in Solution *A*. A total of 25 µL of Solution *A* was mixed with 425 µL of water and then 50 µL of IS was added to it, resulting in Solution *B*. A total of 300 µL of acetonitrile was added to 100 µL of Solution *B*, which resulted in Solution *C*. Solution *C* was vortexed and centrifuged for 5 min at 13,300 rpm and 100 µL was transferred into vials. Then, 5 µL of the sample was injected for the LC-MS/MS analysis.

### 2.6. Method Validation

The analytical method parameters were validated according to the performance criteria defined by the “Bioanalytical Method Validation: Guidance for Industry” (United States Food and Drug Administration) [31], the “Fitness for Purpose of Analytical Methods” (Eurachem) [32] and the “Validation of Analytical Procedures: Text and Methodology” (International Council for Harmonisation) [33].

The method was validated based on linearity, lower limit of detection (LLOD), lower limit of quantitation (LLOQ), accuracy, precision, matrix effect and stability. Linearity was measured using a 5-point calibration curve for each AA in 9 replicates (3 parallel sample preparation on each of the 3 validation days). LLOD and LLOQ values were evaluated for each AA. Due to the lack of AA-free real blank matrix, these values were identified from pure water; however, the working concentration of AAs in the biological samples was higher even after the dilution steps during sample preparation. LLOD and LLOQ were defined as the lowest concentration with a signal-to-noise ratio greater than 3 and 10, respectively. Further criteria for LLOQ were that the accuracy and precision values must be lower than 20%. Accuracy (relative error, RE%) and precision (relative standard deviation, RSD%) were assessed via an analysis of the calibration standards within the same day (3 parallel sample preparation) and on 3 different validation days; overall, 9 measured values were studied. The matrix effect was studied by comparing the LC-MS/MS chromatograms, the peak intensities and the slopes of the calibration curves of AAs with three different dilution solvents (water, Hank’s Balanced Salt Solution (HBSS) and serum). The biological serum samples were frozen when arrived in the laboratory within 1 week and were prepared within a day. The post-preparative stability of calibration points was performed under short-term stability at 10 °C for 3 h and long-term stability at 5 °C for 24 h. Our goal was to measure the biological samples immediately; therefore, we did not perform a stability test longer than 1 day.

## 3. Results and Discussion

### 3.1. MS Optimization

Although there are several optimized MRM parameters in the literature, including transitions and voltages, we decided to conduct automatic MRM optimization by infusing the individual AA stock solutions to find the most intense fragment ions for each AA, where the background was minimal and no interference was detected. Our goal was to use MRM transitions capable of distinguishing isobaric AAs. The MRM transitions were also optimized for cysteine and cystine, but their concentration can vary significantly due to environmental conditions (sample collection, storage and preparation). Since the Department of Pediatrics (where we got the samples from) does not measure these amino acids from serum samples as the concentration of these molecules is not related to diseases, we decided to omit them from our validated method.

In the case of AAs having a molecular weight below *m*/*z* 100 (Gly and Ala), due to the low fragmentation efficiency, only one significant fragment ion was formed and used for quantitation. The classical fragment-based transition was used as a quantifier and the pseudo-MRM (where the precursor ion mass is set as fragment mass with low collision energy) as a qualifier transition in the case of these two AAs.

Ile/Leu and Lys/Gln are known as isobaric AAs. We used an additional and specific MRM transition to distinguish the two isobaric molecules, in addition to their different retention time. There are AAs having only one Da difference in their masses of protonated ions, like Leu (132 *m*/*z*), Asn (133 *m*/*z*) and Asp (134 *m*/*z*), or Gln (147 *m*/*z*) and Glu (148 *m*/*z*). To distinguish these ions, different MRM transitions and differences in retention times were used.

Since the source parameters, such as spray voltage, source-gas values and temperature, play a key role in ion formation, our previously optimized conditions [25] were slightly modified to reach the highest intensity for the AAs. The ion optics parameters, such as declustering potential, entrance potential, collision energy and cell exit potential (DP, EP, CE and CXP, respectively), were optimized for each AA. The optimized values are summarized in Table 1.

### 3.2. HPLC Method Development

Different chromatographic conditions (stationary and mobile phases) were tested to find the optimal conditions for separation and quantitation of very polar AAs. The HILIC technique is suggested for the separation of polar compounds, where a normal-phase column is used with polar mobile phases, usually modified with buffers. Therefore, different HILIC columns (SeQuant^®^ ZIC^®^-HILIC, Luna HILIC) and normal-phase columns (Ascentis Express RP-Amide, Luna NH2) were tested with different mobile phase combinations (water, 10 mM ammonium formate in water, acetonitrile, methanol, and methanol containing formic acid in 0.1 *v*/*v*%), but none of them was robust and accurate enough for the separation of AAs. Many molecules eluted with poor retention, separation could not be achieved, and the peak symmetry and shape were not sufficient enough. An Rx-SIL normal-phase silica column was used in our previous work for the separation and quantitation of free nucleic acid bases, so we decided to try that column for AAs. This column resulted in excellent retention and separation and good peak shape for all AAs. The Rx-SIL column is a totally porous silica column capable of separating basic, neutral and acidic molecules as well, which helps to retain and separate AAs with different structures and polarities. This column is compatible with all common mobile phases. It is important to mention that the usage of ammonium ions as a classical modifier in HILIC eluents is not recommended as they can modify the Rx-SIL silica column surface, resulting in asymmetric broad analyte peaks, and can reduce the column lifetime. In our experiments, water and acetonitrile, both containing 0.1 *v*/*v*% formic acid, were used as eluents. These are the most commonly used eluents in reversed-phase chromatography, where acetonitrile is the stronger eluent. However, in the case of normal-phase chromatography, the more polar solvent is the stronger, the concentration of which increases during the gradient. Formic acid (as a mobile-phase modifier) controls the pH of the mobile phase. At low pH, the overall charge of amino acids is positive, which is very important for HILIC separation.

We tried several fine-tuning in the gradient profile to reach the best separation. A short isocratic part followed by a segmented gradient increasing the aqueous eluent from 25% to 45%, and then to 80%, was found to be the best. The segmented gradient was necessary for better separation of AAs. The gradient part of the chromatography method takes 7 min, and then the program includes the washing and re-equilibration part. However, the retention time of some amino acids was longer than 7 min. Due to the gradient delay, which was exactly 1 min, all components were eluted from the column even before the start of the washing part. The washing and equilibrating regions were part of the method and were set to obtain the initial conditions back. The overall runtime of the method was 15 min including the equilibration time. This gradient profile resulted in the best separation and peak shapes for AAs. The injected volume was set to 5 µL because the AAs in the samples were in relatively high concentrations, and no larger injected volume was required.

**Figure 1:**
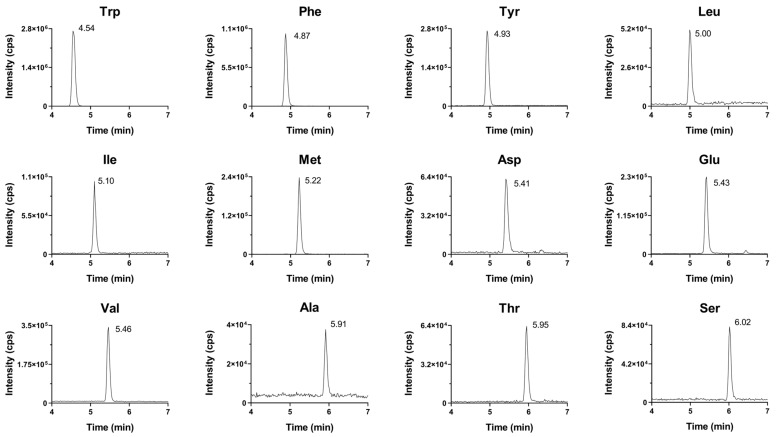

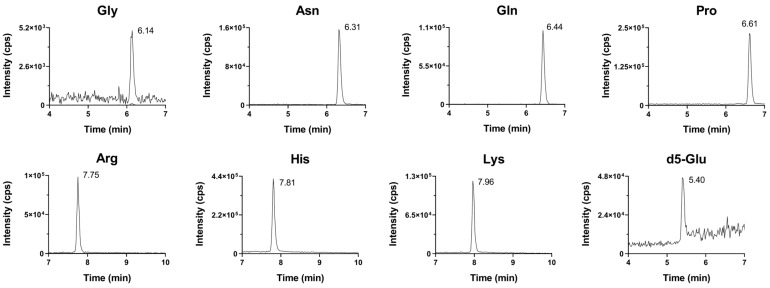
Extracted ion chromatograms for all AAs and the internal standard at a concentration of 50 nM.

The chromatograms obtained are shown in Figure 1. Complete baseline separation cannot be achieved for all components; however, the mass spectrometer can give further selectivity by measuring the individual mass-to-charge ratios of the ions in the MRM chromatograms.

In contrast with the classical HILIC methods found in the literature for the quantitation of underivatized amino acids, we used a totally porous silica normal-phase column with commonly used reversed-phase eluents. In our method, the preparation of the mobile phases is much simpler: no weighting of buffer salts and no pH adjustment is required, and only formic acid should be pipetted to the eluents. This chromatographic method uses simple and commonly used eluents with a very robust column, which enables reproducible retention times. The components are eluted during the gradient part of the method, and they have sufficient retention, while the program is faster than most of the other methods in the literature.

### 3.3. Development of Sample Preparation

AAs are polar and water-soluble compounds, so distilled water was used for the dilution steps. After dilution, the internal standard was added to the diluted serum samples. The added concentration was set to reach a final 50 nM concentration for the sample to be suitable for injection. Finally, proteins had to be precipitated prior to chromatographic injection. Methanol and acetonitrile were tested for protein precipitation, and acetonitrile was found to give higher sensitivity. The peak areas for many amino acids were higher, the background was lower and, therefore, the signal-to-noise ratios also improved. Finally, 100 µL of diluted serum sample and 300 µL of acetonitrile were mixed and centrifuged for 5 min at 13,300 rpm. The final 1:3 sample/acetonitrile volume ratio resulted in the same solvent composition for injection as the initial gradient in the LC method. An overall 800-fold dilution was performed on the initial biological sample during the sample preparation protocol before LC-MS/MS analysis.

### 3.4. Set-up of the Quantitation Model

We wanted to create a fast and reliable quantitation method with simple and relatively cheap sample preparation. We decided to reduce the isotope-labeled standards and use just one internal standard to develop a cost-effective method. Two candidates were tested, 4-aminobutyric acid-2,2-d2 (d2-GABA) and L-glutamic acid-2,3,3,4,4-d5 (d5-Glu). d2-GABA was used previously in our laboratory, so we selected this isotope-labeled standard for the tests. Finally, d5-Glu was selected because its retention time is nearly in the middle of the chromatographic elution of AAs. In the case of d5-Glu, a better peak shape was obtained, so we used d5-Glu for further measurements.

**Figure 2:**
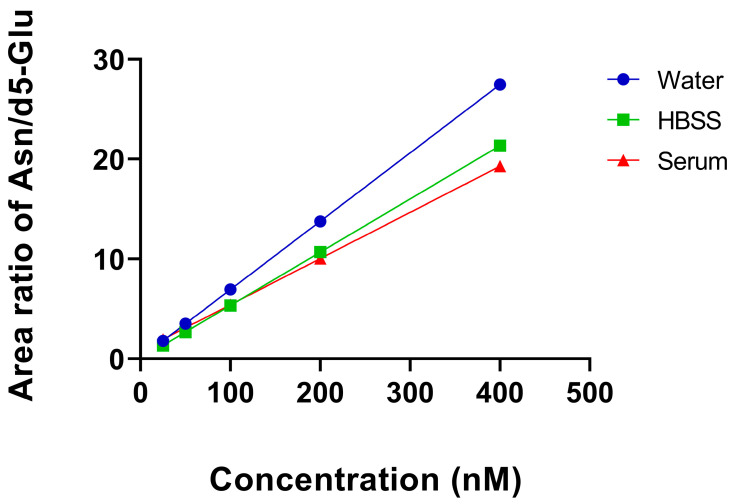
Calibration curves for Asn: concentration vs. peak area of Asn normalized with d5-Glu internal standard. Five parallel measurements were achieved.

Because there are no real blank serum samples for AAs, we decided to compare three models to obtain information about the matrix effect and precision. Calibration points were prepared at different concentration levels from pure solvent (water), the model matrix (HBSS) and the biological samples, and the signals were compared in the function of concentration. A decrease in signals was observed in the case of the model matrix and biological samples compared to the pure solvent. It is due to the matrix effect present in the biological samples. The matrix effect was significantly decreased but still present after diluting the prepared samples 10 times. Further dilution of the samples (preparation of calibration standards, Section 2.4) gave better peak shapes and baseline; however, the slopes of the calibration curves were slightly different from that obtained from the biological samples. A much stronger matrix effect could be detected in the standard addition model of the serum samples compared to that in aqueous calibration. As a representative example, the changes in the slopes of the calibration curves for asparagine in different models are shown in Figure 2. From these data, we concluded that neither the pure solvent nor the model matrix calibration gave accurate results regarding the real concentration of the AAs. Standard addition proved to be the only precise method for analyzing AAs in serum samples.

Finally, we decided to use the standard addition model to quantitate the concentration of AAs in the biological serum samples. The preparation of the calibration points is described in Section 2.4. The matrix is identical in the case of each calibration point and the real samples as well.

**Figure 3:**
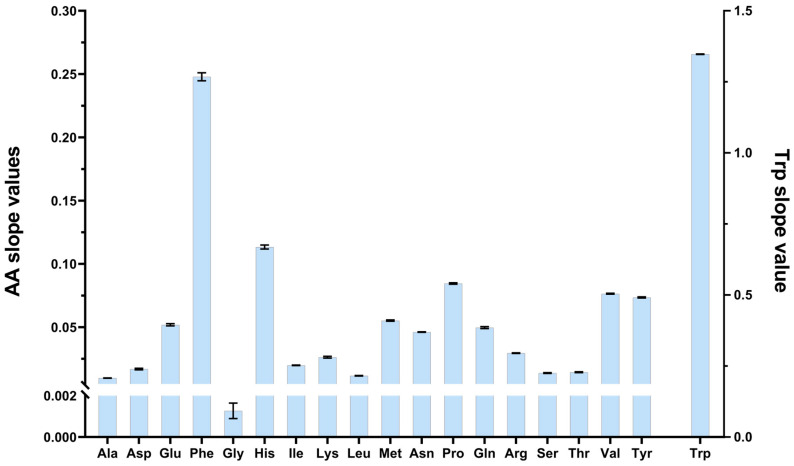
Summary of the parallel calibration measurements. The mean values of the slopes of AAs are shown on the left *Y*-axis, except Trp. The value of Trp is shown on the right *Y*-axis. The standard deviation was plotted for all AAs as error bars.

The slopes of the calibration curves of the individual AAs, obtained from five different serum samples and prepared on the same experimental day, were compared and found to be very similar. The results are shown in Figure 3. The average value of the slopes was plotted for every AA. It can be seen that the slopes are well reproducible while having only a small standard deviation for all individual AAs. This finding can simplify the application of the addition calibration model, while no addition should be performed on each biological sample to obtain the concentration of individual samples. It is enough to use one sample to perform addition at different (five) levels, and the calibration curve obtained can be used for all the samples measured on the same batch with the same biological matrix. This simplified calibration model produced accurate and reliable quantitation data for all of the AAs examined.

### 3.5. Method Validation

#### 3.5.1. Linearity and Detection Limit

Calibration solutions were prepared from one serum sample by adding the AA working solutions (1, 2, 4, 8 and 16 µM, respectively) and internal standard (2 µM) to each. The solutions were parallel prepared on the same day and on three different days, so nine replicate datasets were available. The final AA concentration of the five calibration points was 25, 50, 100, 200 and 400 nM, respectively, and 50 nM for the internal standard. This standard addition range was wide enough to obtain a noticeable increase for every AA concentration. A calibration curve is a plot of the concentration-and-area ratio of the analyte and the internal standard, where weighting is 1/concentration for AAs. The calibration curves fitted well to the calibration points over the entire concentration range, and the correlation coefficient (R) values were above 0.994 for each AA.

LLOD and LLOQ values were identified from pure water and became part of the validation process; however, the concentrations of AAs in the biological samples were much higher even after the dilution process. The analytical method parameters, such as LLOD, LLOQ, the fitted calibration equation and R values, are summarized in Table 2.

Linear regression gave good accuracy (relative error, RE) and precision (relative standard deviation, RSD) for the concentration of each AA at each concentration level (Table S1).

#### 3.5.2. Stability

The human serum samples were kept at −20 °C until they arrived in the laboratory for measurement. The samples were prepared and measured within a day. Human serum samples are stable below −18 °C for up to a year [34], so only post-preparation stability experiments were performed. The original serum samples were divided into aliquots, and these aliquots were separately thawed and used for the validation experiments. The stability test was achieved with n = 9 replicate injections. Our results showed that the calibration points were stable in the short term. The prepared samples could be kept safely in the cooled autosampler or at 5 °C for 1 day.

### 3.6. Application of the Method on Biological Samples

The newly developed and validated method was applied to biological samples. The AA values were measured on 12 randomly taken serum samples. They were collected from non-metabolic and inherited metabolic disorder patients and were anonymized before arrival. Our laboratory had no information about the patients and the samples. The results were not used for clinical treatment.

Three parallel sample preparations were performed on the same day, and each sample was measured three times. A summary of the measurements (mean ± relative standard deviation) is shown in Table S2. To calculate the original concentration of the samples (µM), we have to consider the sample dilution (800-fold) during the sample preparation protocol.

The parallel measurements resulted in great analytical precision, and the RSD values were lower than 10%. Based on the concentration ranges (for children and adults) according to the University of California San Francisco (2019) [35], most of the AA values are within the healthy range. In addition, our results are in good concordance with a commercially available kit [36] used by laboratory specialists for diagnostic testing, where the serum samples arrived from. However, some samples’ values are significantly out of the healthy values for some AAs, which may be caused by different health statuses.

## 4. Conclusions

A rapid and sensitive LC-MS/MS method was developed and validated for the quantitation of natural, underivatized AAs from serum samples. Compared to the existing methods, our new method represents a reliable and simpler alternative solution for quantitation of underivatized AAs via LC-MS/MS. The separation of AAs was based on HILIC mechanism using a normal-phase HPLC column by using classical reversed-phase eluents in the gradient mode. The overall runtime was 15 min. A simple sample preparation was applied consisting of only dilution by water, the addition of the internal standard to the serum samples and protein precipitation with acetonitrile. The advantage of this sample preparation method is that it does not require time-consuming derivatization steps and is not expensive, as there is no need for complex commercial kits and only one isotope-labeled internal standard is needed to be used. The method resulted in sufficient retention of very polar components, while the peak shapes and the retention times were reproducible. The method is very robust; hundreds of injections were measured during method development and validation without peak distortion, while detecting the same retention time for each component. The standard addition method was used for the most precise quantitation of AAs from serum samples. Parallel measurements gave good reproducibility, and the slopes of the calibration curves for the same amino acid were found to be similar. This simplified quantitation, as there was no need for standard addition for every biological sample, and the calibration curve obtained from one serum sample could be used for other samples on the same day.

This method can be further improved by the usage of an UHPLC column instead of a normal HPLC column. We used a relatively high flow rate, causing large eluent consumption, but in the HILIC mode, the larger part of the eluents is water.

The validated method was successfully applied to the study of serum samples. We could recognize differences in their concentrations; however, no further consequences could be determined without knowing the details of the patients’ status. The developed method is simpler, faster and more accurate compared to alternative methods found in the literature, and it is easily adaptable in clinical laboratories. It can also be a good alternative to commercially available kits.

## Figures and Tables

**Table 1:** Optimized MS/MS conditions for AAs.

AA and Abbreviation		MRM Transition (*m*/*z*)	Declustering Potential (DP, V)	Entrance Potential (EP, V)	Collision Energy (CE, eV)	Cell Exit Potential (CXP, V)
Alanine	Ala	90 → 90	26	6.5	7	4
		90 → 44	21	10.5	17	4
Aspartic acid	Asp	134 → 74	30	10	15	5
		134 → 88	30	10	25	5
Glutamic acid	Glu	148 → 84	30	10	20	5
		148 → 130	30	10	15	5
Phenylalanine	Phe	166 → 120	30	10	15	15
		166 → 103	30	10	15	15
Glycine	Gly	76 → 30	30	10	15	15
		76 → 76	30	10	15	15
Histidine	His	156 → 110	30	10	20	5
		156 → 83	30	10	30	5
Isoleucine	Ile	132 → 86	30	10	15	5
		132 → 69	30	10	21	5
		132 → 44	30	10	15	5
Lysine	Lys	147 → 84	1	10	21	14
		147 → 130	1	10	13	16
		147 → 41	1	10	41	8
Leucine	Leu	132 → 86	30	10	15	5
		132 → 44	1	10	29	20
		132 → 43	1	10	33	20
Methionine	Met	150 → 104	30	10	15	15
		150 → 56	30	10	20	15
Asparagine	Asn	133 → 87	30	10	13	5
		133 → 74	30	10	19	5
Proline	Pro	116 → 70	30	10	20	15
		116 → 68	30	10	30	15
Glutamine	Gln	147 → 84	1	10	13	8
		147 → 130	1	10	23	12
		147 → 56	1	10	39	26
Arginine	Arg	175 → 70	30	10	20	5
		175 → 116	30	10	30	5
Serine	Ser	106 → 60	30	10	15	15
		106 → 42	30	10	25	15
Threonine	Thr	120 → 74	30	10	15	15
		120 → 56	30	10	20	15
Valine	Val	118 → 70	30	10	15	15
		118 → 55	30	10	30	15
Tryptophan	Trp	205 → 188	30	10	15	15
		205 → 146	30	10	20	15
Tyrosine	Tyr	182 → 136	30	10	15	15
		182 → 91	30	10	15	15
Internal Standard						
Glutamic acid-d5	d5-Glu	153 → 135	11	10	13	14
		153 → 88	11	10	21	8

**Table 2:** Summary of the analytical parameters of linear regression, including values of LLOD and LLOQ for AAs. (R: correlation coefficient, LLOD: lower limit of detection, LLOQ: lower limit of quantitation).

Amino Acid	Regression Equation	R	LLOD (nM)	LLOQ (nM)
Ala	y = 0.010x + 3.98	0.9998	25	50
Asp	y = 0.017x + 0.69	0.9998	5	10
Glu	y = 0.051x + 18.39	0.9999	1	5
Phe	y = 0.249x + 27.14	0.9996	0.5	1
Gly	y = 0.001x + 0.36	0.9998	25	50
His	y = 0.112x + 10.72	0.9999	5	10
Ile	y = 0.020x + 1.81	0.9999	1	5
Lys	y = 0.027x + 5.40	0.9994	1	5
Leu	y = 0.012x + 1.90	0.9998	1	5
Met	y = 0.056x + 2.69	0.9999	1	5
Asn	y = 0.046x + 0.78	0.9999	5	10
Pro	y = 0.084x + 17.88	0.9995	5	10
Gln	y = 0.049x + 4.08	0.9999	1	5
Arg	y = 0.030x + 3.60	0.9998	5	10
Ser	y = 0.014x + 3.12	0.9997	10	25
Thr	y = 0.014x + 2.46	0.9998	10	25
Val	y = 0.076x + 18.73	0.9998	1	5
Trp	y = 1.348x + 35.40	0.9997	0.1	0.5
Tyr	y = 0.074x + 10.68	0.9995	1	5

## Data Availability

Data is contained within the article or Supplementary Material.

## Supplementary Materials

The following supporting information can be downloaded at: https://www.mdpi.com/article/10.3390/cimb45120586/s1, Figure S1: Extracted ion chromatograms for all AAs and the internal standard at the standard addition concentration of 50 nM and the total ion chromatogram of the blank sample (ACN); Table S1: Statistical evaluation of calibration data of AAs in calibration standards. (RE: relative error, RSD: relative standard deviation), Table S2: Application of the method on 12 human serum samples. Results of the parallel measure-ments with mean concentration (μM) and RSD values (%) are shown. (RSD: relative standard devi-ation). Healthy range concentrations (children and adults) based on the concentration ranges according to the University of California San Francisco (2019).
