# Supplementary material for: Application of Normal-Phase Silica Column in Hydrophilic Interaction Liquid Chromatography Mode for Simultaneous Determination of Underivatized Amino Acids from Human Serum Samples via Liquid Chromatography–Tandem Mass Spectrometry
Source: Curr Issues Mol Biol. 2023 Nov 22;45(12):9354–67. doi: 10.3390/cimb45120586 (PMC10741747; doi:10.3390/cimb45120586)
Supplement: Supplementary file 1 [file cimb-45-00586-s001.zip › cimb-2699402-supplementary.pdf]

current issues in molecular biology

MDPI

Supplementary Information

Application of Normal Phase Silica Column in Hilic Mode for Simultaneous Determination of Underivatized Amino Acids from Human Serum Samples by Liquid Chromatography–Tan-dem Mass Spectrometry

Krisztina Németh [1,2], Ildikó Szatmári [3], Viktória Tőkési [3] and Pál T. Szabó [1,*]

1. MS Metabolomics Research Laboratory, Centre for Structural Science, Research Centre for Natural Sci-ences, Hungarian Research Network, Magyar tudósok krt. 2, H-1117 Budapest, Hungary
2. Institute of Chemistry, Eötvös Loránd University, Pázmány Péter u. 1/A, H-1117 Budapest, Hungary
3. Department of Pediatrics, Semmelweis University, Bókay János u. 54, H-1083 Budapest, Hungary

* Correspondence: szabo.pal@ttk.hu

Table S1. Statistical evaluation of calibration data of AAs in calibration standards. (RE: relative error, RSD: relative standard deviation).

Amino acid	Theoretical concentration														
	25 nM			50 nM			100 nM			200 nM			400 nM		
	Mean	RE%	RSD%	Mean	RE%	RSD%	Mean	RE%	RSD%	Mean	RE%	RSD%	Mean	RE%	RSD%
Ala	25.8	3.2	3.5	50.1	0.2	1.6	96.5	3.5	1.3	202.2	1.1	0.8	400.8	0.2	0.4
Asp	25.8	3.1	2.3	49.2	1.6	1.1	96.8	3.2	0.4	199.5	0.2	0.6	403.4	0.9	0.7
Glu	25.7	2.8	2.0	48.4	3.2	0.4	100.5	0.5	0.4	199.3	0.3	0.5	401.1	0.3	0.5
Phe	25.7	2.9	3.1	47.6	4.7	3.3	103.1	3.1	1.0	196.4	1.8	1.8	401.6	0.4	1.3
Gly	23.9	4.6	4.4	51.8	3.6	1.6	102.1	2.1	1.9	198.2	0.9	0.5	399.4	0.2	0.5
His	25.0	0.2	1.4	49.8	0.4	0.6	101.6	1.6	0.8	199.1	0.5	0.7	400.1	0.0	0.5
Ile	24.7	1.1	1.9	50.2	0.3	0.4	100.9	0.9	1.5	200.6	0.3	0.4	399.1	0.2	1.0
Lys	25.5	2.2	1.6	51.0	2.1	2.5	95.8	4.2	0.7	195.8	2.1	3.5	404.2	1.1	1.6
Leu	25.1	0.3	1.1	49.6	0.7	0.6	101.5	1.5	3.8	198.9	0.6	2.7	400.4	0.1	0.6
Met	25.1	0.4	3.3	50.0	0.0	2.1	99.7	0.3	1.6	199.3	0.3	1.1	400.9	0.2	0.9
Asn	24.9	0.5	1.1	49.9	0.2	1.4	100.3	0.3	1.2	201.6	0.8	0.3	398.2	0.4	0.8
Pro	24.7	1.3	4.0	49.7	0.6	6.1	101.4	1.3	1.7	202.9	1.5	1.6	392.7	1.8	2.7
Gln	25.0	0.1	0.9	50.0	0.0	2.0	100.3	0.3	2.3	199.4	0.3	1.6	400.2	0.1	0.7
Arg	25.1	0.3	0.0	50.7	1.3	0.7	98.3	1.7	2.8	197.0	1.5	1.9	403.6	0.9	0.5
Ser	24.0	4.1	0.6	51.3	2.6	1.6	103.1	3.1	2.0	197.1	1.5	0.6	399.5	0.1	1.1
Thr	25.7	2.8	1.1	48.4	3.3	3.7	99.9	0.1	2.8	200.7	0.3	1.2	399.9	0.0	0.5
Val	25.8	3.3	0.3	48.7	2.7	2.6	99.7	0.3	1.9	199.3	0.4	1.7	401.7	0.4	0.8
Trp	25.4	1.4	3.6	48.9	2.1	1.8	99.9	0.1	3.0	204.0	2.0	1.6	397.3	0.7	0.5
Tyr	24.7	1.4	0.6	50.6	1.2	3.2	101.6	1.6	1.6	193.7	3.2	3.3	402.4	0.6	2.2

Table S2. Application of the method on 12 human serum samples. Results of the parallel measurements with mean concentration (µM) and RSD values (%) are shown. (RSD: relative standard deviation). Healthy range concentrations (children and adults) based on the concentration ranges according to the University of California San Francisco (2019).

AA	Healthy range	Concentration (mean ± RSD% µM)											
		S1	S2	S3	S4	S5	S6	S7	S8	S9	S10	S11	S12
Ala	200 – 510	346.8 ± 9.3	302.2 ± 6.7	220.5 ± 0.6	265.6 ± 9.5	270.5 ± 9.7	138.9 ± 7.9	892.5 ± 5.7	164.0 ± 1.7	244.6 ± 6.5	155.1 ± 3.0	219.5 ± 8.9	170.4 ± 3.2
Asp	0 – 26	43.3 ± 3.7	27.1 ± 0.5	39.0 ± 5.4	32.5 ± 3.0	117.1 ± 2.0	29.1 ± 2.8	119.7 ± 2.5	40.9 ± 9.7	25.1 ± 2.5	30.2 ± 2.1	28.2 ± 5.0	33.1 ± 8.4
Glu	18 – 140	316.8 ± 3.8	109.4 ± 1.1	144.2 ± 0.1	126.3 ± 7.7	224.1 ± 0.6	152.4 ± 2.7	516.0 ± 5.3	222.5 ± 8.8	128.0 ± 1.0	123.1 ± 1.5	209.3 ± 7.1	166.7 ± 3.4
Phe	26 – 86	111.3 ± 4.9	100.4 ± 3.5	93.7 ± 1.7	85.3 ± 3.8	126.5 ± 3.0	78.9 ± 0.7	305.7 ± 4.8	80.8 ± 4.5	81.5 ± 0.3	74.3 ± 0.7	85.8 ± 6.1	68.4 ± 1.3
Gly	110 – 330	422.3 ± 3.7	364.7 ± 8.9	375.4 ± 8.0	361.0 ± 6.5	858.7 ± 7.3	267.7 ± 4.8	492.2 ± 0.9	306.7 ± 3.1	454.6 ± 2.5	345.5 ± 2.0	287.8 ± 6.9	319.4 ± 3.6
His	26 – 120	121.4 ± 3.7	98.5 ± 3.0	83.1 ± 5.8	98.8 ± 4.9	136.6 ± 0.4	85.4 ± 2.0	133.8 ± 2.9	117.0 ± 3.1	103.4 ± 1.9	95.6 ± 3.4	96.4 ± 3.3	96.7 ± 7.9
Ile	37 – 140	116.4 ± 7.1	100.2 ± 1.8	59.1 ± 3.6	77.7 ± 1.4	129.6 ± 2.0	62.5 ± 4.6	151.8 ± 2.8	87.7 ± 4.6	95.6 ± 1.0	58.3 ± 2.2	74.6 ± 7.1	57.0 ± 7.0
Lys	120 – 290	458.1 ± 2.0	222.4 ± 1.8	200.6 ± 4.0	218.0 ± 4.1	258.5 ± 2.7	170.9 ± 1.1	598.8 ± 6.7	207.3 ± 8.1	160.5 ± 1.9	178.1 ± 4.2	259.5 ± 4.3	272.3 ± 8.4
Leu	66 – 140	236.4 ± 1.3	185.0 ± 6.4	125.4 ± 5.2	151.2 ± 2.8	233.0 ± 2.7	130.1 ± 1.5	345.9 ± 2.7	181.2 ± 2.8	156.3 ± 0.8	120.3 ± 1.0	140.6 ± 8.6	112.0 ± 5.3
Met	13 – 30	38.8 ± 3.6	28.9 ± 1.2	16.1 ± 3.0	21.1 ± 6.1	31.8 ± 1.6	15.7 ± 0.4	25.2 ± 5.3	16.9 ± 6.7	26.4 ± 3.0	17.2 ± 0.4	39.5 ± 5.2	36.7 ± 3.0
Asn	15 – 130	16.6 ± 7.2	11.7 ± 2.0	14.4 ± 5.5	11.4 ± 1.6	17.3 ± 7.1	10.4 ± 1.0	31.4 ± 4.7	11.1 ± 2.3	12.7 ± 0.1	10.2 ± 4.4	13.0 ± 7.1	10.4 ± 4.7
Pro	110 – 360	272.2 ± 4.6	306.5 ± 2.7	151.1 ± 2.5	152.2 ± 3.3	265.3 ± 1.3	134.2 ± 1.2	353.6 ± 3.8	107.1 ± 4.8	197.3 ± 1.1	125.5 ± 0.3	173.7 ± 6.6	159.5 ± 5.0
Gln	390 – 730	128.2 ± 3.5	89.7 ± 3.1	52.8 ± 2.6	71.1 ± 2.7	54.2 ± 1.9	71.7 ± 1.5	222.5 ± 2.4	60.4 ± 4.9	81.4 ± 0.5	73.4 ± 0.7	127.7 ± 7.5	98.7 ± 6.2
Arg	13 – 120	129.3 ± 4.8	129.5 ± 1.3	223.4 ± 2.4	155.6 ± 3.3	121.0 ± 0.1	51.3 ± 3.7	96.3 ± 0.6	158.1 ± 10.0	101.6 ± 3.6	115.6 ± 2.9	172.3 ± 0.1	242.1 ± 9.3
Ser	56 – 150	220.9 ± 5.3	192.2 ± 0.4	146.2 ± 5.9	152.5 ± 1.2	660.4 ± 0.3	129.5 ± 3.6	169.6 ± 4.2	182.6 ± 3.1	179.2 ± 1.2	159.6 ± 0.7	169.9 ± 6.5	168.0 ± 2.3
Thr	67 – 240	173.2 ± 0.2	112.3 ± 3.5	68.0 ± 5.7	88.6 ± 4.7	174.6 ± 2.6	48.6 ± 5.0	± 65.6 ± 2.9	70.8 ± 3.7	104.4 ± 0.1	77.7 ± 1.4	103.5 ± 9.3	154.9 ± 5.1
Val	26 – 110	240.9 ± 3.8	317.2 ± 2.2	192.5 ± 4.0	245.2 ± 3.8	345.1 ± 3.2	177.8 ± 0.3	443.0 ± 3.9	283.6 ± 4.3	228.5 ± 3.4	214.9 ± 0.1	159.6 ± 7.7	166.1 ± 2.7
Trp	3.1 – 8.3	6.8 ± 1.2	11.7 ± 4.6	9.7 ± 2.3	12.1 ± 7.9	13.0 ± 6.2	9.9 ± 2.6	15.7 ± 2.7	9.4 ± 4.5	11.1 ± 3.0	8.7 ± 3.6	13.6 ± 10.0	11.0 ± 4.1
Tyr	26 – 110	149.7 ± 2.2	114.7 ± 6.3	66.8 ± 3.6	85.1 ± 7.9	153.5 ± 3.4	80.5 ± 1.0	134.4 ± 5.1	56.5 ± 9.5	76.0 ± 0.4	59.8 ± 0.5	132.3 ± 6.2	118.7 ± 0.2

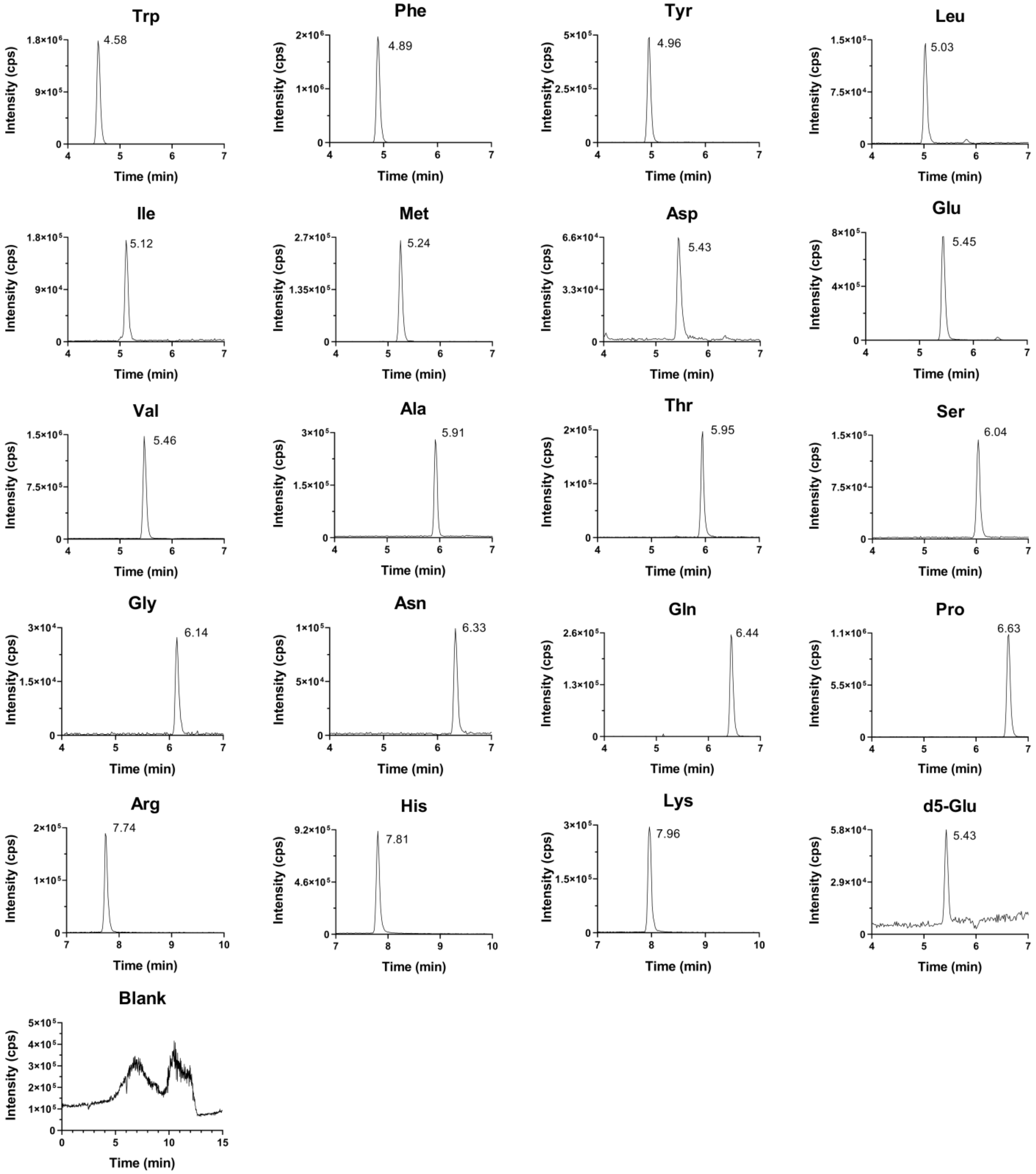

Figure S1. Extracted ion chromatograms for all AAs and the internal standard at the standard addition concentration of 50 nM and the total ion chromatogram of the blank sample (ACN).
